# Comprehensive reconstruction and evaluation of *Pichia pastoris* genome-scale metabolic model that accounts for 1243 ORFs

**DOI:** 10.1186/s40643-017-0152-x

**Published:** 2017-05-09

**Authors:** Rui Ye, Mingzhi Huang, Hongzhong Lu, Jiangchao Qian, Weilu Lin, Ju Chu, Yingping Zhuang, Siliang Zhang

**Affiliations:** State Key Laboratory of Bioreactor Engineering, East China University of Science and Technology, No.130, Meilong Road, Shanghai, 200237 China

**Keywords:** Genome-scale metabolic model, *Pichia pastoris*, Multi-omics, β-Galactosidase

## Abstract

**Background:**

*Pichia pastoris* is one of the most important cell factories for production of industrial enzymes and heterogenous proteins. The genome-scale metabolic model of high quality is crucial for comprehensive understanding of the *P. pastoris* metabolism.

**Methods:**

In this paper, we upgraded *P. pastoris* genome-scale metabolic model based on the combination of latest genome annotations and literatures. Then the performance of the new model was evaluated using the Cobra Toolbox v2.0.

**Results:**

Compared with the recently published model iMT1026, the reaction number in the new model iRY1243 was increased from 2035 to 2407 and the metabolite number was increased from 1018 to 1094. Accordingly, the unique ORF number was increased from 1026 to 1243. To improve the metabolic functions of *P. pastoris* genome-scale metabolic model, the biosynthesis pathways of vitamins and cofactors were carefully added. iRY1243 showed good performances when predicting the growth capability on most of the reported carbon and nitrogen sources, the metabolic flux distribution with glucose as a sole carbon source, the essential and partially essential genes, and the effects of gene deletion or overexpression on cell growth and *S*-adenosyl-l-methionine production.

**Conclusion:**

iRY1243 is an upgraded *P. pastoris* genome-scale metabolic model with significant improvements in the metabolic coverage and prediction ability, and thus it will be a potential platform for further systematic investigation of *P. pastoris* metabolism.

**Electronic supplementary material:**

The online version of this article (doi:10.1186/s40643-017-0152-x) contains supplementary material, which is available to authorized users.

## Background

*Pichia pastoris* (*P. pastoris*), one important cell factory, has been widely used to express more than 200 kinds of industrial enzymes and heterogenous proteins (Cereghino and Cregg 2000; Ahmad et al. 2014) because of its many interesting properties: the availability of well-established protocols and techniques for gene operation (Bhataya et al. 2009; Nocon et al. 2014; Morozkina et al. 2016), the ability of post-translational modification (De Schutter et al. 2009), the ease of establishing high-cell density culture on simple and defined media, and the simple separation and purification processes (Macauley-Patrick et al. 2005).

To improve the protein production levels by *P. pastoris*, comprehensive understanding of its metabolism is necessary. Genome-scale metabolic model (GSMM) has been one of the most widely used tools in system biology (Kim et al. 2012) and has shown its potential for predicting cell phenotypes under certain conditions (Famili et al. 2003; Fong et al. 2003), integrating multiple omic data (Saha et al. 2014; Angione et al. 2016), discovering metabolic network characteristics (Feist and Palsson 2008), and predicting engineering target for improving the titer of industrial cell factories.

The earliest metabolic network model of *P. pastoris* is the small model of central carbon metabolism network model (Maaheimo et al. 2001), which is used to calculate the ^13^C metabolic flux distribution (Sola et al. 2004). The gene sequencing of *P. pastoris* has laid the foundation for GSMM construction (Mattanovich et al. 2009; Kuberl et al. 2011). There are different *P. pastoris* GSMM models. Sohn et al. (2010), Chung et al. (2010), and Caspeta et al. (2012) established the PpaMBEL1254, iPP668, and iLC915 models, respectively. The model iMT1026 was reconstructed by merging PpaMBEL1254, iPP668, and iLC915 models, and adding the synthesis and decomposition pathway of fatty acid, sphingolipid biosynthesis pathway, oxidative phosphorylation, and glycosylation pathway (Tomas-Gamisans et al. 2016). Compared with the latest *Saccharomyces cerevisiae* GSMMs, the quality of these *P. pastoris* models still needs to be improved at least in three aspects: the balance of each reaction in mass and electrical charge, the model metabolic coverage, and the correction in GPRs. The biomass components of *P. pastoris* (Carnicer et al. 2009) and the utilization of energy (Chung et al. 2010) have been reported. With the availability of new gene annotations and literatures about *P. pastoris*, it is necessary to update the *P. pastoris* GSMM models to improve the model performance.

In this paper, based on the latest gene annotations and the newly published literatures, we reconstructed a new genome-scale metabolic network model of *P. pastoris*. The new model iRY1243 contained 1243 annotated genes, 2407 reactions, 1740 metabolites, and 9 reaction locations. At the same time, we updated the cell components of *P. pastoris* based on the latest literatures. The model performance was evaluated by the data from RNA-Seq, chemostat experiments, and ^13^C labeling experiments. Model iRY1243 was then used to predict the secretion of products and essential genes on synthetic medium.

## Methods

### Procedures for model reconstruction

**Fig. 1:**
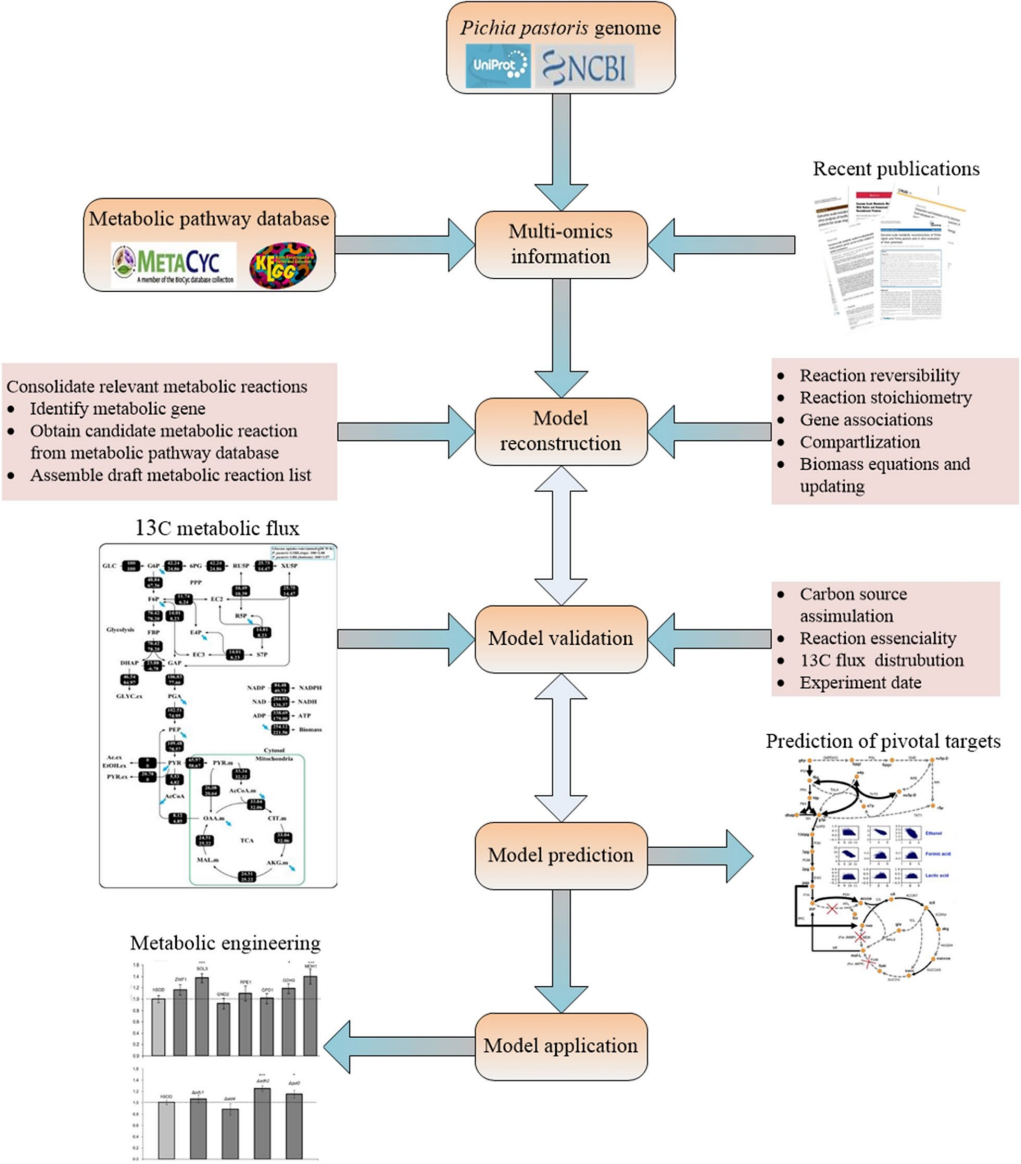
Workflow for reconstruction of *P. pastoris* GSMM iRY1243

The procedures for reconstruction of *P. pastoris* GSMM could be divided into two phases (Fig. 1). Firstly, by collating and evaluating the existing models, we upgraded the GSMM based on the existing iPP668, PpaMBEL1254, iLC915, and iMT1026 models and re-annotated the information of all metabolites by referring to the databases of KEGG and BiGG, to ensure that each metabolite had correct structure and charge in pH 7.2. Then, each reaction in models was manually checked and corrected, thus making it comparable with the published GSMM model.

Subsequently, to update the *P. pastoris* GSMM systematically, the genome annotation information from mainly three databases (KEGG, UniProtKB, IMG) was gathered and merged (Table 1). To establish the new gene–protein–reaction associations (GPRs), the genes were collected from each database. With the aid of KEGG and Enzyme databases, the relationship between proteins and reactions were further checked to ensure the high quality of GPRs in the new *P. pastoris* GSMM. Next, using the gapFind function of the Cobra Toolbox v2.0 to identify dead-end metabolites, essential reactions from KEGG and other databases were added to reduce the number of dead-end metabolites to improve the function of the metabolic network.

**Table 1:** Information of databases used in this study

Abbreviation	Full name	Purpose	Link
KEGG	Kyoto encyclopedia of genes and genomes	Obtain genome annotation, reaction information chemical compounds annotation	http://www.genome.jp/dbget-bin/www_bfind?compound
CheBI	Chemical entities of biological interest	Obtain chemical compounds annotation	https://www.ebi.ac.uk/chebi/
BiGG	Biochemical genetic and genomic knowledgebase	Obtain reaction information and chemical compounds annotation	http://bigg.ucsd.edu/bigg/main.pl
Ensembl fungi	Ensembl fungi	Obtain genome annotations	http://fungi.ensembl.org/index.html
UniProtKB	The universal protein resource knowledgebase	Obtain genome annotations	http://www.uniprot.org/
IMG	The integrated microbial genomes system	Obtain genome annotations	http://img.jgi.doe.gov/
ENZYME	Enzyme nomenclature database	Obtain relations between enzyme and reactions	http://enzyme.expasy.org/

Lastly, the cell components and the energy parameter were updated. Compared with the primordial model, some new components of cell, like the cofactors including folate and thiamin, were added to ensure the function of the corresponding metabolic pathways. With the fitting of the data from chemostat cultivations, the non-growth-associated maintenance (NGAM) in the model was adjusted from 3.7 to 2.52 mmol ATP/gDCW h.

### Experimental procedures

#### Strains

The *Pichia pastoris* strains GS115 and G1HL were used to evaluate the model performance.

#### Medium and culture conditions

In this study, 50 mL YPG medium as the seed medium was taken in 500-mL flask (yeast extract 10%, peptone 20%, glycerol 20%). The composition of the medium used in chemostat cultivations was as follows: 10 g/L glucose, 9.1 g/L K2SO4, 0.46 g/L CaSO4, 7.5 g/L MgSO4·7H2O, 2.06 g/L KOH, 13.4 mL/L H3PO4, and 6 mL/L PTM1. The components of PTM1 were described by Baumann et al. (2008).

The culture conditions for seed culture and chemostat culture were described by Nie et al. (2014). Chemostat cultivation was carried out in a 5-L bioreactor (National Engineering Research Center for Biotechnology, Shanghai, China) with a working volume of 3 L. With glucose as the sole carbon source, the initial aeration rate was set to 1 vvm, controlled by a mass flow controller (Sevenstar, Beijing, China). The dissolved oxygen (DO) was maintained above 30% by adjusting the agitation and the aeration to ensure fully aerobic conditions. The O_2_ and CO_2_ concentrations in the off-gas were measured online using a process mass spectrometer (MAX300-LG, Extrel, America). Pressure, pH, and temperature were controlled at 0.04 MPa, pH 5.5 (with adding NH_3_·H_2_O), and 30 °C, respectively. The specific growth rate of the cells was controlled by the dilution ratio (D).

### Determination of the concentration of cell, glucose, and other extracellular metabolites

The cell concentration was measured from the optical density at 600 nm. For dry cell weight (DCW) measurement, 10 mL broth was filtered using a pre-dried and weighted microporous membrane (Shanghai Diqing Filtration Technology CO., LTD Shanghai, China). After being washed 3 times, the filter paper was dried at 105 °C for 3 h. The glucose kits (Shanghai Kexin Biotechnology Institute, China) were used for residual glucose concentration analysis. Extracellular metabolite measurements (such as ethanol, acetic acid, propionic acid, formic acid, pyruvic acid, and acetaldehyde) were performed as described in Nie et al. (2014).

### Sensitivity analysis

During the sensitivity analysis of the GSMM, the specific glucose consumption rate was changed from 0 to 2.0 mmol/gDCW h. In each simulation, only one of the following model parameters was evaluated, including the lipid composition (6.2–16.2%), protein composition (37–47%), RNA composition (6.6–16.6%), DNA composition (0.1–1.0%), carbohydrate composition (26.9–36.9%), and NGAM value (1.26–3.78 mmol ATP/gDCW h). The specific biomass growth rate and oxygen uptake rate were simulated to evaluate the effects of the changes of parameters on the model prediction ability.

### Calculation of energy parameters

The ATP required for cell survival can be calculated by chemostat experiments. The generated ATP can be divided into two forms: non-growth-associated ATP maintenance (NGAM) and growth-associated ATP maintenance (GAM). GAM required for biomass synthesis (i.e., precursor biosynthesis and polymerization) can be obtained from literature. NGAM, used for cell maintenance, is an independent reaction in the model, which can be calculated from chemostat data.

NGAM and GAM are added to calculate the total amount of ATP required for cell growth, as shown in the following equation:

$$r_{\text{ATP}} = Y_{{x{\text{ATP}}}} * \mu + m_{\text{ATP}},$$

where *Y*_*x*ATP_ and *m*_ATP_ represent the GAM and NGAM, respectively, *μ* is the specific growth rate, and *r*_ATP_ represents the total amount of ATP consumed by *P. pastoris*.

### Biomass composition

As the biomass composition has obvious effects on the model validation and the strain improvement, we updated the cell composition of *P. pastoris*. The biomass is composed of macromolecular substances (i.e., protein, lipid, DNA, RNA, carbohydrates, small-molecule pool). The details on the biomass components of *P. pastoris* were referred from Verduyn et al. (1991), Carnicer et al. (2009), De Schutter et al. (2009) and Tomas-Gamisans et al. (2016). The detailed information of biomass composition is described in the supporting information (see Additional file 2).

### Constraint-based FBA

Constraint-based flux-balanced analysis (FBA) (Bordbar et al. 2014) was widely used in the reconstruction of GSMM model, analysis of strain capabilities under different environmental and genetic perturbations, and prediction of strain phenotype (Orth et al. 2010). Generally, FBA predicts metabolic fluxes based on maximization of objective functions (typically the specific growth rate or the target metabolite production rate) (Feist and Palsson 2010; Sanchez et al. 2012), and the basic framework of FBA is composed of variables, objectives, and constraints. The constraints used in FBA include the balance of intracellular metabolites (Eq. 2), the reversibility and the demarcation line of reactions, as well as the extracellular metabolite exchange rates (Eq. 3). In this study, all FBA was conducted with the cell growth rate as the objective function (Eq. 1). Each reaction had an upper and a lower bound on the flux it can carry. For reversible reaction, the upper and lower bounds were set at −1000 and 1000, respectively, while for irreversible reaction the lower bound was set at 0. FBA was conducted using Cobra Toolbox v2.0 (Schellenberger et al. 2011) and Gurobi 6 solver based on Matlab.

1 $${\text{Objective}}{:} \, \hbox{max} /\hbox{min} \, Z = C^{T} *v$$

2 $${\text{Constraints}}{:} \, S*v = 0$$

3 $${\text{lb }} \le \, v \, \le {\text{ ub}},$$

where *S* is a matrix of m*n and *m* and *n* are the numbers of metabolites and reactions, respectively. In Eq. 1, *C* ^*T*^ represents the coefficient of metabolites in the objective function. In Eq. 3, *v* is the rate of all reactions. lb and ub defined the lower and upper bounds of the flux for each reaction, respectively.

During the simulation with iRY1243, NGAM and GAM were set at 2.52 mmol ATP/gDCW h and 20.4 mmolATP/gDCW, respectively (see Additional file 3). The lower bound of exchange reactions except for glucose, ammonia, oxygen, sulfur, phosphorus, and Fe^2+^ was set at 0. To predict the specific growth rate (*μ*), the exchange rates of glucose were set at −0.478, −0.692, −0.942, −1.294, and −1.846 mmol/gDCW h, respectively. The exchange rates of other substrates and by-products were set at the corresponding values at each dilution rate.

### Prediction of growth-supporting carbon and nitrogen sources

To investigate the prediction capability of iRY1243, 30 carbon and 21 nitrogen sources for *P. pastoris* were collected. FBA was used to analyze the growth capability on each carbon or nitrogen source. For prediction of carbon utilization, only NH_4_^+^ was set as the nitrogen source, while the phosphorus and sulfur sources were maintained sufficient. At the same time, the metabolites of the other exchange reactions containing carbons were set as zero. For prediction of nitrogen utilization, glucose was used as the only carbon source. The target carbon or nitrogen source was considered growth supporting if the predicted specific growth rate was above zero.

### Prediction of essential genes

The prediction of essential genes and partially essential genes was conducted using the singleGeneDeletion function based on Cobra Toolbox. During the essential gene analysis, according to the matrix of GPRs and the Boolean rule definition, the fluxes of reactions containing the relating genes were set at zero, while the bounds of other reactions were maintained constant. Based on the simulated specific growth rate, the genes were classified into essential genes (the calculated specific growth rate was 0), partially essential genes (the calculated specific growth rate of *P. pastoris* was in the range from 0 to the maximum specific growth rate), and non-essential genes (the calculated specific growth rate was the same as the maximum specific growth rate). The synthetic medium, which was made up of glucose, oxygen, ammonia, sulfur, and phosphorus, was used to predict the essential genes for *P. pastoris* growth.

### Transcriptome analysis

The strain *P. pastoris* G1HL was used. The medium and procedures for seed culture and chemostat cultivations were described in Nie et al. (2014) with glucose as the only carbon source.

During the stable phase of *P. pastoris* G1HL culture, about 20 mL broth was taken into three 50-mL centrifuge tubes. Samples were centrifuged at 4000 rpm for 10 min at 4 °C and supernatant was discarded. The harvested cells were washed three times with 0.9% NaCl and dried with a filter paper. The samples were stored in a −80 °C refrigerator. The RNA extraction and sequence, read mapping, quantification, and data analysis were accomplished by KangChen Bio-tech Inc. (Shanghai, China).

## Results and discussion

### Reconstruction of the *P. pastoris* GSMM iRY1243

The update of the *P. pastoris* GSMM was based on the iPP668, PpaMBEL1254, iLC915, and iMT1026 models. The procedure for reconstruction of *P. pastoris* is shown in Fig. 1. First, the elements and charge in all reactions were balanced based on the metabolites’ annotation information. Gap analysis was also conducted and gaps were reduced by adding essential reactions from KEGG and other databases. To overcome the deficiencies of these models, *P. pastoris* gene annotation information from three common databases (KEGG, UniProtKB, and IMG) was sorted and compared. Among them, KEGG was the largest metabolic network database.

### Comparison of iRY1243 with the other models

As shown in Table 2, iRY1243 contains 2407 reactions and 1740 metabolites, which is more than that in the model of iMT1026 (Tomas-Gamisans et al. 2016), indicating that this upgrade enlarged the scale of the *P. pastoris* GSMM. The ORF number in iRY1243 is 1243, which is also more than that in iMT1026. Based on the latest gene annotations, the model iRY1243 contains more genes compared to the previous model iMT1026, such as the genes relating to the metabolism of porphyrin and chlorophyll, biotin, the membrane transport reactions, etc. The biomass contains the products of these pathways, which are crucial for the prediction of essential genes. iRY1243 is an upgraded *P. pastoris* GSMM with significant improvements in the metabolic coverage. In conclusion, the *P. pastoris* GSMM was comprehensively improved based on gene annotations from different databases and literatures (Additional file 1).

**Table 2:** Comparison among different GSMMs of *P. pastoris*

	iPP668	PpaMBEL1254	iLC915	iMT1026	iRY1243
Genes	668	540	915	1026	1243
Metabolites	1177	1058	1302	1689	1740
Reactions	1354	1254	1423	2035	2407
Cytosolic	623	604	790	1059	1316
Mitochondrial	163	155	205	268	313
Peroxisomal	66	66	64	102	102
Extracellular	12	11	0	16	5
Endoplasmic reticulum	15	7	34	41	43
Golgi apparatus	4	8	4	13	14
Vacuolar	3	6	12	9	10
Nuclear	16	17	0	17	17
Intercompartmental/transport	452	328	314	510	540

Accurate cell composition and NGAM value were important for improving the metabolic functions of GSMM. By referring to the latest GSMM of *P. pastoris* and *Saccharomyces cerevisiae*, the essential cell trace components were added, including the small-molecule pool (see Additional file 2). The NGAM was calculated by chemostat data (see Additional file 3).

### Validation of GSMM

#### Model verification by RNA-Seq

**Fig. 2:**
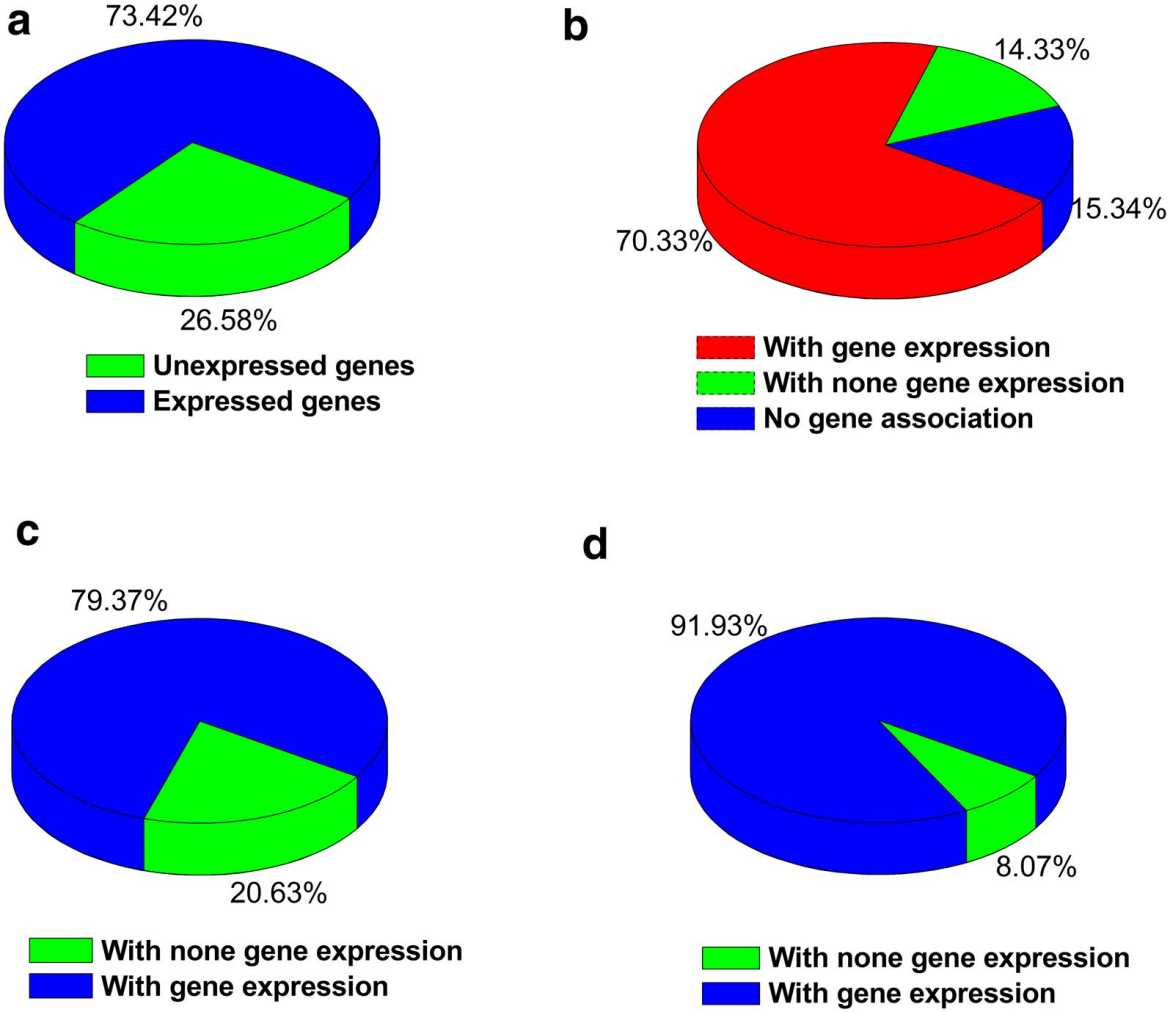
Verification of iRY1243 by RNA-seq data. Proportions of expressed and unexpressed genes to total genes involved in iRY1243 (**a**), proportion of verified reactions, unverified reactions, and no-gene annotation reactions in remained 1784 reactions (removing the exchange and transport reactions) (**b**), proportion of single-gene reactions with and without gene expression (**c**), and proportion of multi-gene reactions with and without gene expression (**d**)

The gene in the newly updated *P. pastoris* GSMM iRY1243 was firstly verified with the latest RNA sequence data. The RNA-Seq results showed that the expression of 2393 genes could be determined in the sampling condition. The iRY1243 contains 1243 genes and the expression of most genes (895 genes, 73.42%) could be verified according to the results of RNA-seq (Fig. 2a) when glucose was used as the sole carbon source. Removing the exchange reactions, among the remaining 1773 reactions, the expression of genes from 14.33% (254 reactions) of these reactions was not measured (Fig. 2b). After removing the exchange reactions and reactions without the corresponding annotated genes, there are 1042 and 471 reactions associated with single and multiple genes, respectively. Based on the transcriptome analysis, about 79.37% of the single-gene reactions and 91.93% of the multi-gene reactions were verified (Fig. 2c, d), indicating that the existence of most reactions in the model iRY1243 was reliable.

#### Sensitivity analysis of iRY1243

**Fig. 3:**
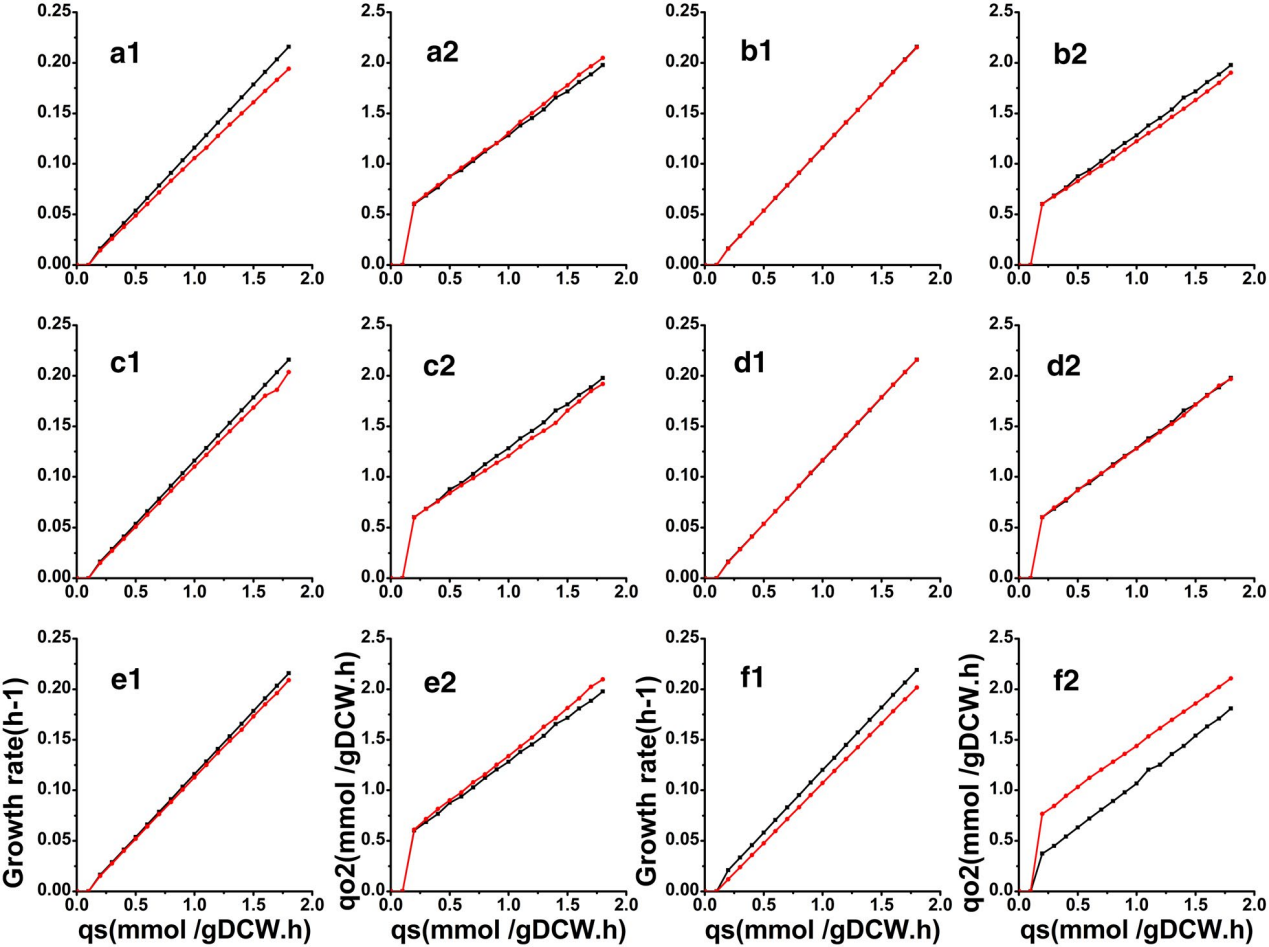
Effects of each parameter change on specific growth rate and specific oxygen uptake rate for sensitivity analysis with iRY1243. The simulations were performed by varying the lipid content (6.2–16.2%) (**a1**, **a2**), protein content (37–47%) (**b1**, **b2**), RNA content (6.6–16.6%) (**c1**, **c2**), DNA content (0.1–1%) (**d1**, **d2**), carbohydrate content (26.9–36.9%) (**e1**, **e2**), and the NGAN (1.26–3.78 mmol ATP/gDCW h) (**f1**, **f2**). *Red* represents the simulated results for the maximum values of the input parameters, and *black* represents the lower content in all cases

During sensitivity analysis, the influence of different biomass components (lipid, protein, RNA, DNA, carbohydrate) and the energy parameters (NGAM) on the prediction accuracy of the strain phenotype was comprehensively investigated. As shown in Fig. 3, the specific growth rate (*µ*) and the specific oxygen uptake rate (qo2) were hardly affected by the changes of the protein, RNA, DNA, and carbohydrate contents. However, as the content of lipid increased, *µ* decreased. The reason may be that the synthesis of fatty acids requires more ATP and NADPH. Different from protein, RNA, DNA, carbohydrate, and the predicted *µ* and qo2 were more obviously affected by NGAM changes. As NGAM increased, the predicted *µ* decreased rapidly, while the qo2 increased accordingly as shown in Fig. 3f.

### Model verification by growth capabilities on different carbon and nitrogen sources

To comprehensively evaluate the prediction ability of the model iRY1243, *P. pastoris* phenotype data reported from 1998 to 2017 were collected (Menendez et al. 1998; Hsieh et al. 2010; Klompmaker et al. 2010; Sohn et al. 2010; Zhang et al. 2015), together with physiological data from our lab. *P. pastoris* could grow on a series of carbon sources and nitrogen sources. The growth-supporting 30 carbon and 21 nitrogen sources can be found in Tables 3 and 4. The in silico growth capabilities of *P. pastoris* on these carbon and nitrogen sources were checked using iRY1243. As shown in Tables 3 and 4, growth on 30 carbon and 21 nitrogen sources could be predicted using iRY1243. Compared to model iMT1026 (Chung et al. 2010; Sohn et al. 2010; Caspeta et al. 2012; Nocon et al. 2014; Irani et al. 2016; Tomas-Gamisans et al. 2016), the prediction ability of iRY1243 was better.

**Table 3:** Evaluation of growth capabilities of *P. pastoris* on different carbon sources (+ for growth and − for non-growth)

Carbon sources	In vivo	iMT1026	iRY1243
Xylose	+	+	+
Glucose	+	+	+
Glutamate	+	+	+
Fumarate	+	+	+
Glycogen	+	+	+
Mannose	+	+	+
Citrate	+	+	+
d-xylose	+	+	+
Acetate	+	+	+
Alanine	+	+	+
Mannitol	+	+	+
Sorbitol	+	+	+
Maltose	+	−	+
Glycerol	+	+	+
Methanol	+	+	+
Fructose	+	+	+
Maltotriose	+	+	+
Trehalose	+	+	+
Ethanol	+	+	+
l-Aspartate	+	+	+
d-Galactose	+	−	+
l-arabinose	+	−	+
Starch	+	−	+
l-rhamnose	+	+	+
Phenylalanine	+	+	+
Pyruvate	+	+	+
Ribose	+	+	+
Succinate	+	+	+
Xylitol	+	+	+
α-Ketoglutarate	+	+	+

**Table 4:** Evaluation of growth capabilities of *P. pastoris* on different nitrogen sources (+ for growth and − for non-growth)

Nitrogen sources	In vivo	iMT1026	iRY1243
Alanine	+	+	+
Ammonia	+	+	+
Phenylalanine	+	−	+
Leucine	+	−	+
Arginine	+	+	+
Aspartate	+	+	+
Glutamate	+	+	+
Asparagine	+	+	+
Citrulline	+	−	+
4-Aminobutanoate	+	+	+
Glutamine	+	+	+
Glycine	+	+	+
Ornithine	+	+	+
Proline	+	+	+
Serine	+	+	+
Threonine	+	+	+
Tryptophan	+	+	+
Tyrosine	+	+	+
Urea	+	+	+
Xanthin	+	+	+
Putrescine	+	+	+

### Model verification by physiological growth parameters

**Fig. 4:**
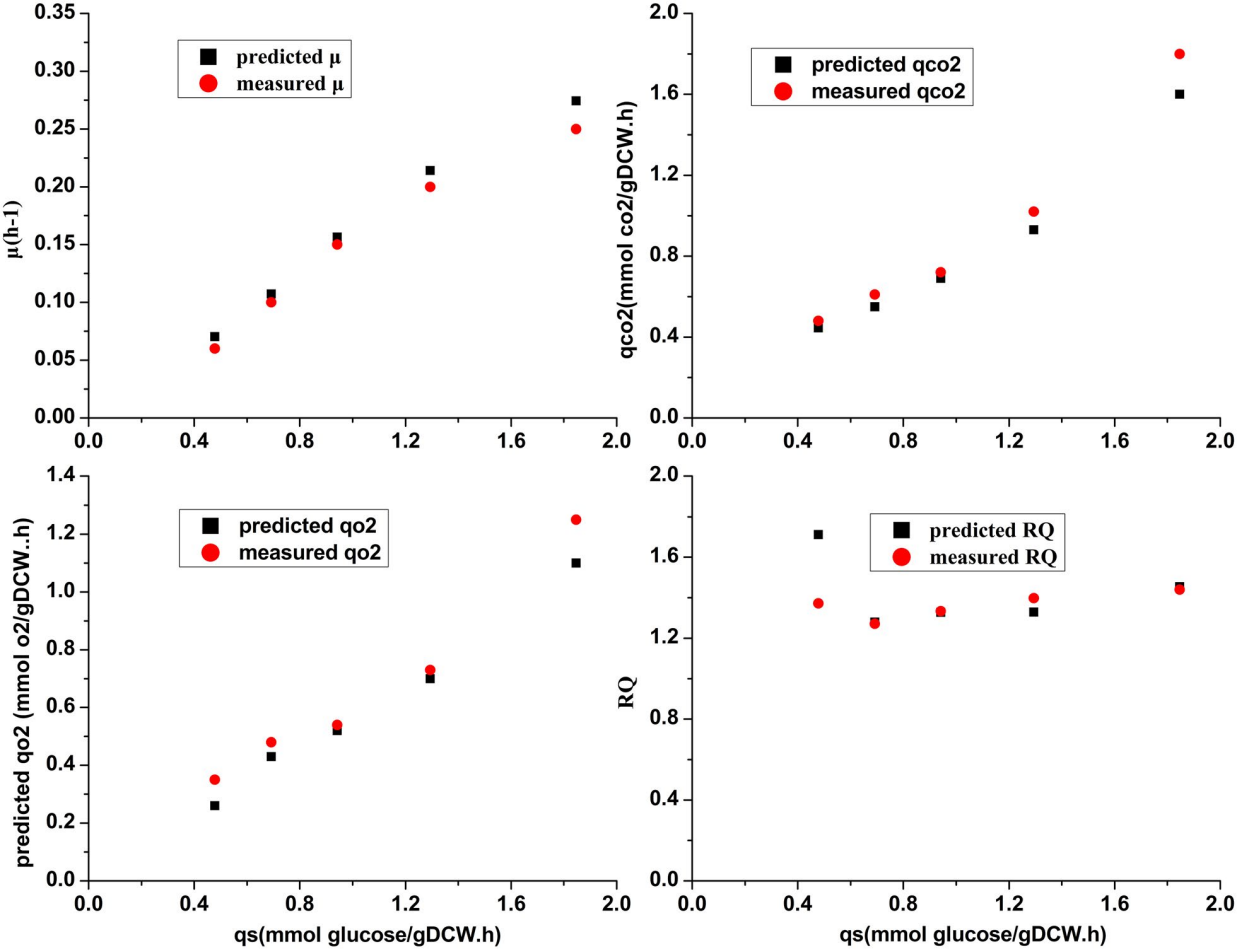
Predicted and measured *μ*, qo2, qco2, and RQ for chemostat cultivation of *P. pastoris* G1HL producing β-galactosidase. *Blue* represents the GSMM simulated results, and *red* represents the experimental data

The maximization of cell growth rate usually served as the objective function in FBA. Thus, a comparison between the measured and predicted *μ* values could show the quality of iRY1243. The reported NGAM of *P. pastoris* was 2.26 mmolATP/gDCW h (Chung et al. 2010), while, according to our calculation based on ^13^C labeled experiment, the NGAM was 9.48 mmolATP/gDCW h for *P. pastoris* G1HL (Nie et al. 2014). Due to the limitations of the central carbon metabolic network model used to calculate the fluxes of pathways, the model cannot contain all the ATP reactions. As the NGAM had an obvious effect on the specific growth rate, the NGAM in the iRY1243 was firstly calculated using *P. pastoris* G1HL chemostat cultivation data without by-product formation. We further compared the predicted and measured values with a series of chemostat cultivations using *P. pastoris* G1HL producing beta-galactosidase with the new NGAM value (2.52 mmol ATP/gDCW h). As shown in Fig. 4, iRY1243 could predict *μ*, qo2, qco2, and RQ under a range of the specific glucose consumption rate inputs.

To compare the function of each model, the chemostat data published by Carnicer et al. (2012) were used to verify the predictive function of the models (see Additional file 4). FBA was used to compare the predictive ability of each model with the maximization of cell growth rate as the objective function. The results showed that iRY1243 and iMT1026 could predict *P. pastoris* specific growth rate and CO2 specific consumption rate. However, the model iMT1026 predicted some unreasonable by-products (citric acid, etc.).

### Model verification by in vivo ^13^C fluxes

**Fig. 5:**
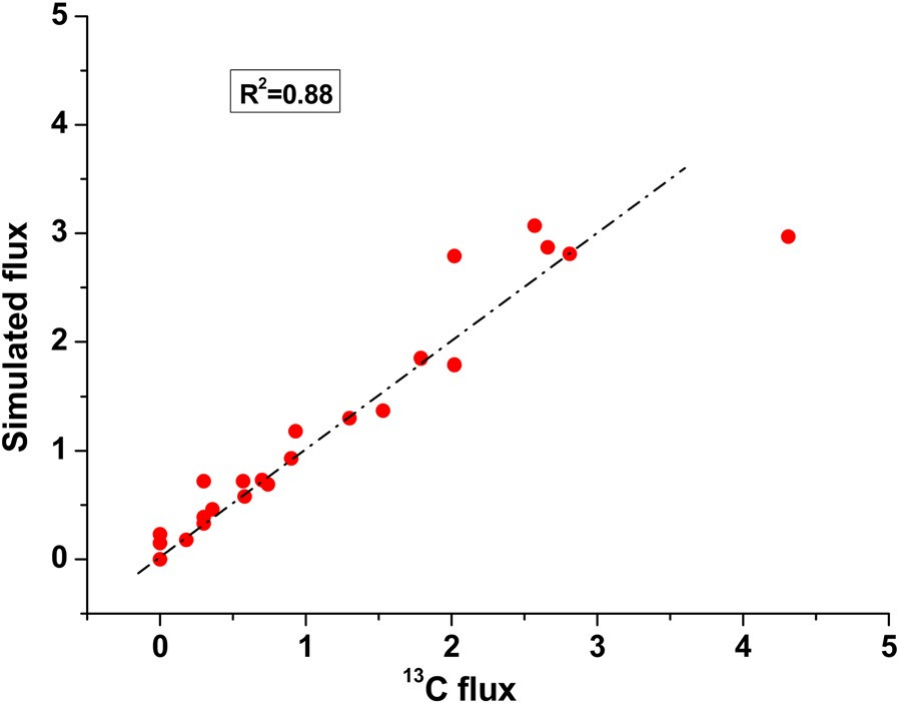
Consistent changes in fluxes can be predicted using iRY1243 compared with ^13^C metabolic fluxes. The growth physiological parameters used for pFBA calculation, as well as the calculated ^13^C fluxes of *P. pastoris* G1HL, were described in previous research (Nie et al. 2014)

To further evaluate the prediction performance of iRY1243, the simulated fluxes using pFBA were compared with the calculated metabolic flux from ^13^C labeled experiments (Nie et al. 2014). As shown in Fig. 5, the correlation coefficient between the simulated fluxes by GSMM and the calculated ^13^C fluxes for G1HL was 0.88, initially showing the good performance of iRY1243. However, there are still significant differences at some points, which may be caused due to the following reasons. Firstly, the algorithms used to calculate the fluxes were totally different between FBA and ^13^C central flux analysis. Secondly, there is a great difference in the size of the two models for the ^13^C flux model only containing the central carbon metabolism pathways. Generally, the ^13^C fluxes were more accurate as there are more constraints from ^13^C labeled metabolite information. On the whole, the high consistency between the simulated fluxes and the calculated ^13^C fluxes validated the good prediction performance of iRY1243. The PPP pathway, EMP pathway, and TCA cycle flux distribution can be well predicted (see Additional file 5).

### Prediction of essential genes

**Fig. 6:**
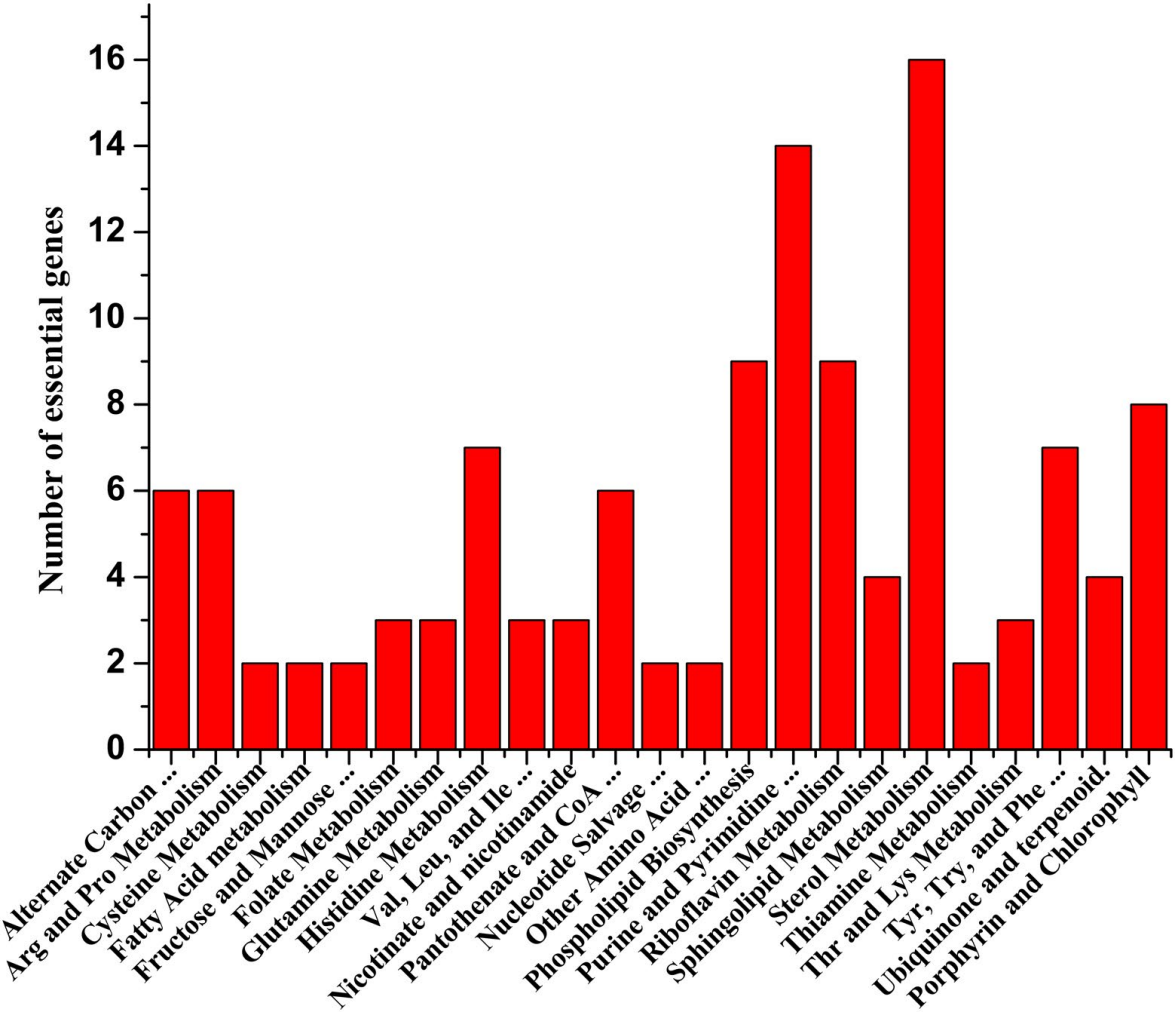
Distribution of essential genes in metabolic subsystems of the *P. pastoris* GSMM

Based on the relationship of genes, proteins, and reactions in the genome-scale metabolic network model, the relationship between genotype and phenotype can be predicted (Thiele and Palsson 2010). The singleGeneDeletion function in Cobra Toolbox v2.0 can be used to predict the essential genes, partially essential genes, and non-essential genes (Schellenberger et al. 2011). On the synthetic medium, 123 essential genes were found, which were related to cofactor metabolism, TCA cycle, lipids biosynthesis, etc. (Fig. 6). At the same time, 169 partially essential genes were obtained (Additional file 6), which may be vital for subsequent in silico strain design (Pan and Hua 2012), considering the fact that the yield of target metabolite production may increase with the slight slowdown of growth.

To further evaluate the potential of the new *P. pastoris* GSMM in rational strain design, data of some engineered *P. pastoris* (the specific growth rate and *S*-adenosyl-l-methionine production) were collected from our lab and previously published studies. Then iRY1243 was used to simulate the effects of the corresponding gene insertion, deletion, or overexpression. The high consistency between the experimental and simulated results (see Additional file 7) indicated that iRY1243 can be used to design a more efficient *P. pastoris* for production of *S*-adenosyl-l-methionine or other heterogenous proteins.

## Conclusion

*Pichia pastoris* GSMM was systematically upgraded based on the latest gene annotations and literatures. The resulting *P. pastoris* GSMM, iRY1243, accounts for 1243 ORFs and contains 2407 reactions and 1094 metabolites. The RNA-Seq data confirmed that the existence of most reactions in iRY1243 was reliable. iRY1243 showed good performance when predicting the cell growth physiological parameters, the growth capability on the reported carbon and nitrogen sources, the metabolic flux distribution with labeled glucose as the sole carbon source, the essential and partially essential genes, and the effects of gene deletion or overexpression on cell growth and *S*-adenosyl-l-methionine production. The significant improvement in the metabolic coverage and prediction ability make iRY1243 a potential platform for further systematic investigation of *P. pastoris* metabolism.

## Additional files

**Additional file 1.** Detailed information of iRY1243.

**Additional file 2.** Biomass composition.

**Additional file 3.** Non-growth-associated ATP maintenance (NGAM) requirement.

**Additional file 4.** Detailed comparison of Pichia pastoris GSMMs prediction performance.

**Additional file 5.** Comparison between ^13^C fluxes and simulated fluxes by iRY1078.

**Additional file 6.** Essential genes and partially essential genes predicted by iRY1078.

**Additional file 7.** Measured and simulated influences of gene deletion or overexpression on the growth and *S*-adenosyl-l-methionine production by *P. pastoris.*

## Abbreviations

*P. pastoris*: *Pichia pastoris*
GSMM: genome-scale metabolic network model
GPRs: gene–protein–reaction associations
NGAM: non-growth-associated maintenance
GAM: growth-associated ATP maintenance
D: dilution ratio
DO: dissolved oxygen
DCW: dry cell weight
FBA: constraint-based flux-balanced analysis
*μ*: specific growth rate
