# Supplementary material for: Comprehensive reconstruction and evaluation of Pichia pastoris genome-scale metabolic model that accounts for 1243 ORFs
Source: Bioresour Bioprocess. 2017 May 9;4(1):22. doi: 10.1186/s40643-017-0152-x (PMC5423920; doi:10.1186/s40643-017-0152-x)
Supplement: Supplementary file 2 — Additional file 2. Biomass composition. [file 40643_2017_152_MOESM2_ESM.docx]

Additional File 2: Biomass composition

**Table 1 Macromolecular composition:**

| **Components** | **g/gDCW** |
| --- | --- |
| Protein | **0.37** |
| RNA | **0.066** |
| DNA | **0.001** |
| Lipids | **0.062** |
| Carbohydrates | **0.369** |
| Small molecules pool | **0.132** |

^a^ The composition is taken from[[1](#_ENREF_1)].

0.370 protein + 0.066 RNA + 0.001 DNA + 0.062 lipid + 0.369 carbohydrate + 0.132 COF => biomass

**Table 2 Proteins composition:**

| **Amino acids** | **mol/mol protein** | **MW (-H2O)** | **g/mol Protein** | **mmol/g Protein** |
| --- | --- | --- | --- | --- |
| Ala | 0.1068 | 71.078 | 7.59 | **0.943** |
| Arg | 0.0674 | 156.186 | 10.53 | **0.595** |
| Asn | 0.0439 | 114.103 | 5.01 | **0.388** |
| Asp | 0.0439 | 115.087 | 5.05 | **0.388** |
| Cys | 0.0015 | 103.143 | 0.15 | **0.013** |
| Gln | 0.0928 | 128.129 | 11.88 | **0.819** |
| Glu | 0.0928 | 129.114 | 11.98 | **0.819** |
| Gly | 0.0712 | 57.051 | 4.06 | **0.629** |
| His | 0.0179 | 137.139 | 2.45 | **0.158** |
| Ile | 0.0412 | 113.158 | 4.66 | **0.364** |
| Leu | 0.0699 | 113.158 | 7.91 | **0.617** |
| Lys | 0.0633 | 128.172 | 8.11 | **0.559** |
| Met | 0.0077 | 131.196 | 1.01 | **0.068** |
| Orn | 0.0153 | 115.154 | 1.76 | **0.135** |
| Phe | 0.0303 | 147.174 | 4.46 | **0.268** |
| Pro | 0.0367 | 97.115 | 3.56 | **0.324** |
| Ser | 0.0643 | 87.077 | 5.60 | **0.568** |
| Thr | 0.0577 | 101.104 | 5.83 | **0.510** |
| Trp | 0.014 | 186.210 | 2.61 | **0.124** |
| Tyr | 0.0213 | 163.173 | 3.48 | **0.188** |
| Val | 0.0558 | 99.131 | 5.53 | **0.493** |

^b^ The composition is taken from [[1](#_ENREF_1)].

^c^ water is substracted from MW to account for water excretion during peptide bond formation.

Protein biosynthesis equation is therefore (in mmol for synthesis of 1 g protein):

0.943 ala-L + 0.595 arg-L + 0.388 asn-L + 0.388 asp-L + 0.013 cys-L + 0.819 glu-L + 0.819 gln-L + 0.629 gly + 0.158 his-L + 0.364 ile-L + 0.617 leu-L + 0.559 lys-L + 0.068 met-L + 0.135 orn + 0.268 phe-L + 0.324 pro-L + 0.568 ser-L + 0.510 thr-L + 0.124 trp-L + 0.188 tyr-L + 0.493 val-L => protein

**Table 3 DNA composition:**

| **DNA** | **MW** | **mol/mol DNA** | **g/mol DNA** | **mmol/gDNA** |
| --- | --- | --- | --- | --- |
| dAMP | 313.2 | 0.2945 | 92.2374 | **0.9534** |
| dCMP | 289.2 | 0.2055 | 59.4306 | **0.6652** |
| dGMP | 329.2 | 0.2055 | 67.6506 | **0.6652** |
| dTMP | 304.2 | 0.2945 | 89.5869 | **0.9534** |

^d^ GC content of Pichia pastoris is about 41.1% [[2](#_ENREF_2)].

^e^ the molecular weight is the weight of the nucleotide monophosphate substracted 1 water, which is lost during esterification

DNA biosynthesis equation is therefore (in mmol for synthesis of 1 g DNA):

0.9534 damp + 0.6652 dcmp + 0.6652 dgmp + 0.9534 dtmp => DNA

**Table 4 RNA composition:**

|  | **mRNA (5%)** | **tRNA(20%)** | **rRNA (75%)** |  |  |  |  |
| --- | --- | --- | --- | --- | --- | --- | --- |
|  | **mol/mol** | **mol/mol** | **mol/mol** | **mol/mol RNA** | **MW q** | **g/mol RNA** | **mmol/g RNA** |
| AMP | 0.2945 | 0.198 | 0.303 | 0.2816 | 328.1982 | 92.4124 | **0.8794** |
| CMP | 0.2055 | 0.342 | 0.227 | 0.2489 | 304.17342 | 75.7164 | **0.7774** |
| GMP | 0.2055 | 0.289 | 0.21 | 0.2256 | 344.1976 | 77.6424 | **0.7045** |
| UMP | 0.2945 | 0.171 | 0.26 | 0.2439 | 305.15814 | 74.4357 | **0.7618** |

^f^ RNA composition was adapted from iMT1026 model[[3](#_ENREF_3)].

RNA biosynthesis equation is therefore (in mmol for synthesis of 1 g RNA):

0.8794 amp + 0.7774 cmp + 0.7045 gmp + 0.7618 ump => RNA

**Table 5 lipids composition:**

| Lipid (LIP) | **g/g LIP** g | **g/g DCW** | **MW** | mmol/g LIP |
| --- | --- | --- | --- | --- |
| Glycerides ^h^ | 0.572 | 0.035 | 779.46 | **0.7272** |
| Sterol | 0.191 | 0.012 | 396.40 | **0.4766** |
| SE | 0.077 | 0.005 | 655.34 | **0.1167** |
| Phospholipids | 0.16 | 0.010 | 772.97 | **0.2051** |
| Sphingolipids | 0.009 | 0.001 | 1016.14 | **0.0091** |

^g^ lipids composition was adapted from iMT1026 model[[3](#_ENREF_3)].

0.2051 PHOSPHOLIPID + 0.4766 sterols + 0.1167 SE + 0.7272 glycerides + 0.0091 SPHLIPID => LIPIDS

**Table 5.1 Glycerides composition:**

| **Glycerides (Glyc)** | **g/g Glyc** | **Core MW** | **Total MW** | **mmol/g Glyc** | **mol/mol Glyc** |
| --- | --- | --- | --- | --- | --- |
| Diacylglycerides | 0.27 | 146.10 | 609.96 | 0.443 | **0.345** |
| Triacylglycerides | 0.73 | 173.10 | 868.89 | 0.840 | **0.655** |

0.345 12dgr_SC + 0.655 triglyc_SC => glycerides

**Table 5.2 Sterols composition:**

| **Sterols** | **μg/mg protein ^k^** | **g/g sterol** | **MW** | **mmol/g sterol** | **mol/mol sterol** |
| --- | --- | --- | --- | --- | --- |
| Zymosterol | 0.700 | 0.037 | 384.638 | 0.095 | **0.0378** |
| Ergosterol | 17.700 | 0.927 | 396.648 | 2.336 | **0.9261** |
| Fecosterol | 0.300 | 0.016 | 398.664 | 0.039 | **0.0156** |
| Episterol | 0.300 | 0.016 | 398.664 | 0.039 | **0.0156** |
| Lanosterol | 0.100 | 0.005 | 426.717 | 0.012 | **0.0049** |

0.0156 epist + 0.9261 ergst + 0.0156 fecost + 0.0049 lanost + 0.0378 zymst => sterols

**Table 5.3 Sphingolipid composition:**

| **Sphingolipid (SPHL)** | **mmol/gDCW ^n^** | **MW** | **g/gDCW** | **mmol/g LIP** | **g/g LIP** | **mol/mol SPHL** |
| --- | --- | --- | --- | --- | --- | --- |
| Ceramide | 0.0000291 | 628.79 | 0.000018 | 0.00047 | 0.00029 | **0.05083** |
| GlcCer | 0.0001260 | 748.20 | 0.000094 | 0.00203 | 0.00152 | **0.22025** |
| mip2c | 0.0001390 | 926.04 | 0.000129 | 0.00224 | 0.00208 | **0.24297** |
| IPC | 0.0001390 | 1099.94 | 0.000153 | 0.00224 | 0.00247 | **0.24297** |
| mipc | 0.0001390 | 1346.34 | 0.000187 | 0.00224 | 0.00302 | **0.24297** |

0.0508 ceravg + 0.2203 GlcCer + 0.243 IPCavg + 0.243 mip2cavg + 0.243 mipcavg => SPHLIPID

**Table 5.4 Steryl Ester composition:**

| **Steryl Ester** | **mmol/mmol SE ^l^** | **MW** | **g/mol** |
| --- | --- | --- | --- |
| Zymosteryl ester | **0.0378** | 643.570 | 24.308 |
| Ergosteryl ester | **0.9261** | 655.580 | 607.153 |
| Fecosteryl ester | **0.0156** | 657.596 | 10.270 |
| Episteryl ester | **0.0156** | 657.596 | 10.270 |
| Lanosteryl ester | **0.0049** | 685.649 | 3.335 |

0.0156 epistest_SC + 0.9261 ergstest_SC + 0.0156 fecostest_SC + 0.0049 lanostest_SC + 0.0378 zymstest_SC => SE

**Table 5.5 Phospholipids composition:**

| **Phospholipids (PL)** | g/g PL ^m^ | MW | Fatty acid residues | **Total MW** | mmol/g PL | mol/mol PL |
| --- | --- | --- | --- | --- | --- | --- |
| Phosphatidate | 2.17 | 224.06216 | 2 | 687.92 | 0.03 | **0.0246** |
| Phosphatidylinositol | 6.73 | 387.2107 | 2 | 851.07 | 0.08 | **0.0617** |
| Phosphatidylserine | 6.41 | 312.14744 | 2 | 776.01 | 0.08 | **0.0645** |
| Phosphatidylcholine | 50.3 | 311.22562 | 2 | 775.09 | 0.66 | **0.5066** |
| Phosphatidylethanolamine | 30.86 | 269.14588 | 2 | 733.01 | 0.43 | **0.3287** |
| Cardiolipin | 2.54 | 506.20346 | 4 | 1433.92 | 0.02 | **0.0138** |
| Lysophospholipids | (1.0) | - | - | - |  |  |
| Dimethylphosphatidylethanolamine | - | - | - | - |  |  |

0.5066 pc_SC + 0.0246 pa_SC + 0.0645 ps_SC + 0.0617 ptd1ino_SC + 0.3287 pe_SC + 0.0138 clpn_SC => PHOSPHOLIPID

**Table 5.6 Fatty acid composition:**

| **Fatty acids (FA)** | **FA** | **g/100 g FA ^i^** | **g/100 g FA ^j^** | **MW** | **mmol/g FA** | **mmol/mmol FA** | **g/mol FA** |
| --- | --- | --- | --- | --- | --- | --- | --- |
| Tetradecanoate | C14:0 | 0.3 | 0.30 | 227.36 | 0.01 | **0.0037** | 0.84 |
| Hexadecanoate | C16:0 | 12.4 | 12.56 | 255.42 | 0.49 | **0.1357** | 34.67 |
| Hexadecenoate | C16:1 | 3 | 3.04 | 253.4 | 0.12 | **0.0331** | 8.39 |
| Octadecanoate | C18:0 | 3.6 | 3.65 | 283.47 | 0.13 | **0.0355** | 10.06 |
| Octadecenoate | C18:1 | 30.5 | 30.90 | 281.45 | 1.10 | **0.3030** | 85.27 |
| Octadecynoate | C18:2 | 27.1 | 27.46 | 279.44 | 0.98 | **0.2711** | 75.76 |
| Octatrienoate | C18:3 | 21.2 | 21.48 | 277.42 | 0.77 | **0.2136** | 59.27 |
| Hexacosanoate | C26 | 0.6 | 0.61 | 395.68 | 0.02 | **0.0042** | 1.68 |
|  | Others | 1.2 | - | - | - | **-** | - |

0.136 hdca + 0.036 ocdca + 0.004 hexc + 0.004 ttdca + 0.033 hdcea + 0.303 ocdcea + 0.271 ocdcya + 0.214 lnlnca <=> fatty

**Table 6 Carbohydrates composition:**

|  | **g/g DCW** | **g/g DCW e** | **g/g CH** | **MW (monomer)** | **mmol/g** Carbohydrates |
| --- | --- | --- | --- | --- | --- |
| Glycogen | 0.106 | 0.106 | 0.287 | 162.1406 | 1.769 |
| Trehalose | 0.003 | 0.003 | 0.008 | 342.29648 | 0.022 |
| Chitin | 0.015 | 0.015 | 0.042 | 203.19256 | 0.205 |
| Mannan | 0.062 | 0.062 | 0.167 | 162.1406 | 1.030 |
| β-D-glucan | 0.077 | 0.183 | 0.497 | 162.1406 | 3.065 |

^h^ Composition taken from [[3](#_ENREF_3)].

Carbohydrates biosynthesis equation is therefore (in mmol for synthesis of 1 g carbohydrates):

1.769 glycogen + 0.022 tre + 0.205 chitin + 1.030 mannan + 3.065 16BDglcn => carbohydrate

**Table 7 Small molecules pool composition:**

For simplification is we assumed that the selected small molecules are equally represented (w/w) in the pool. We include some essential metabolites in the biomass composition so as to qualitative account for the essentiality of their synthesis pathways. The composition of these metabolites is summarized in the following table:

| **Metabolite** | **mmol/gDCW** |
| --- | --- |
| Cyclic-AMP | 0.000001 |
| NAD | 0.000001 |
| NADP | 0.000001 |
| CoA | 0.000001 |
| THF | 0.000001 |
| FMN | 0.000001 |
| FAD | 0.000001 |
| thiamine | 0.000001 |
| ubiquinone | 0.000001 |
| Glutathione | 0.000001 |
| Protoheme | 0.000001 |

^i^ Essential molecules are included in biomass composition in order to consider the essentiality of their biosynthesis pathways. The amount of biomass composition is not quantitative and it is only considered its qualitative contribution.

Small molecules pool biosynthesis equation is therefore (in mmol for synthesis of 1 g SMALL MOLECULES):

0.000001 nad + 0.000001 nadp + 0.000001 coa + 0.000001 thf + 0.000001 fmn + 0.000001 q6 + 0.000001 fad + 0.000001 thm + 0.000001 gthrd + 0.000001 pheme + 0.000001 camp => COF

It is noted that since the contribution of these metabolites is minute, we assume that they do not contribute quantitatively to any calculations with regards to cellular biomass.

**Table 8 Growth associated ATP requirement for polymerization:**

| **Polymer** | **mmol ATP/g polymer** |
| --- | --- |
| Protein | 37.7 |
| Carbohydrate | 12.8 |
| RNA | 26.0 |
| DNA | 26.0 |

^j^ The ATP requirement for polymerization of each species is obtained from [[4](#_ENREF_4)].

# References

[1] Carnicer M, Baumann K, Toplitz I, Sanchez-Ferrando F, Mattanovich D, Ferrer P, Albiol J. Macromolecular and elemental composition analysis and extracellular metabolite balances of Pichia pastoris growing at different oxygen levels. Microbial Cell Factories. 2009, 8.

[2] De Schutter K, Lin YC, Tiels P, Van Hecke A, Glinka S, Weber-Lehmann J, Rouze P, de Peer YV, Callewaert N. Genome sequence of the recombinant protein production host Pichia pastoris. Nature Biotechnology. 2009, 27:561-U104.

[3] Tomas-Gamisans M, Ferrer P, Albiol J. Integration and Validation of the Genome-Scale Metabolic Models of Pichia pastoris: A Comprehensive Update of Protein Glycosylation Pathways, Lipid and Energy Metabolism. Plos One. 2016, 11.

[4] Verduyn C, Stouthamer AH, Scheffers WA, van Dijken JP. A theoretical evaluation of growth yields of yeasts. Antonie Van Leeuwenhoek. 1991, 59:49-63.
