# Supplementary material for: Comprehensive reconstruction and evaluation of Pichia pastoris genome-scale metabolic model that accounts for 1243 ORFs
Source: Bioresour Bioprocess. 2017 May 9;4(1):22. doi: 10.1186/s40643-017-0152-x (PMC5423920; doi:10.1186/s40643-017-0152-x)
Supplement: Supplementary file 3 — Additional file 3. Non-growth-associated ATP maintenance (NGAM) requirement. [file 40643_2017_152_MOESM3_ESM.docx]

Non-growth associated ATP maintenance (NGAM) requirement

The NGAM refers to the amount of ATP required by the cell even when it is not growing. This energy consumed for purposes other than the production of new cell material has been extensively reviewed ([van Bodegom 2007](#_ENREF_3)). In this study, we determined the NGAM requirement for our chemostat experiment using a conventional method of finding the y-intercept of the plot of glucose uptake rate against dilution rate([Pirt 1982](#_ENREF_2)).

By maximizing ATP turnover under the glucose uptake constraint of 1 mmol/gDCW-hr, the ATP yield is evaluated as YATP, max = 21.5 mol ATP/ mol glucose ([Chung, Selvarasu et al. 2010](#_ENREF_1)). Using this value and the y-intercept (0.1174 mmol glucose/gDCW-hr), we can calculate the NGAM requirement to be about 2.52 mmol ATP/gDCW-hr.

# References

1. Chung, B., S. Selvarasu, A. Camattari, J. Ryu, H. Lee, J. Ahn, D. Lee and D.-Y. Lee (2010). "Research Genome-scale metabolic reconstruction and in silico analysis of methylotrophic yeast Pichia pastoris for strain improvement." Microb Cell Fact **9**.
2. Pirt, S. J. (1982). "Maintenance energy: a general model for energy-limited and energy-sufficient growth." Arch Microbiol **133**(4): 300-302.
3. van Bodegom, P. (2007). "Microbial maintenance: A critical review on its quantification." Microbial Ecology **53**(4): 513-523.
