# Supplementary material for: Comprehensive reconstruction and evaluation of Pichia pastoris genome-scale metabolic model that accounts for 1243 ORFs
Source: Bioresour Bioprocess. 2017 May 9;4(1):22. doi: 10.1186/s40643-017-0152-x (PMC5423920; doi:10.1186/s40643-017-0152-x)
Supplement: Supplementary file 4 — Additional file 4. Detailed comparison of Pichia pastoris GSMMs prediction performance. [file 40643_2017_152_MOESM4_ESM.docx]

**All of Phicha pastoris GSMMs prediction performance comparison**

The FBA analysis was used to compare the prediction ability of each model with the maximization of cell growth as the objective function. According to the data reported by Carnicer et al. ([Carnicer, ten Pierick et al. 2012](#_ENREF_1)), we compare the prediction capabilities of each model under different oxygen confinement conditions, as shown in Fig . S5.

Fig . S5. **The comparison of the predicted values with experimental data for each GSMM model.** Graphs with growth rate (A) CO2 production (B) predictions simulating glucose chemostats at different oxygen conditions with glucose, O2 and ethanol fluxes constrained to the experimental values.

# References

1. Carnicer, M., A. ten Pierick, J. van Dam, J. J. Heijnen, J. Albiol, W. van Gulik and P. Ferrer (2012). "Quantitative metabolomics analysis of amino acid metabolism in recombinant Pichia pastoris under different oxygen availability conditions." Microbial Cell Factories **11**.
