# Supplementary material for: Comprehensive reconstruction and evaluation of Pichia pastoris genome-scale metabolic model that accounts for 1243 ORFs
Source: Bioresour Bioprocess. 2017 May 9;4(1):22. doi: 10.1186/s40643-017-0152-x (PMC5423920; doi:10.1186/s40643-017-0152-x)
Supplement: Supplementary file 5 — Additional file 5. Comparison between 13C fluxes and simulated fluxes by iRY1078. [file 40643_2017_152_MOESM5_ESM.docx]

**^13^C flux compared with GSMM iRY1078 simulated flux**

**Fig . S6 ^13^C flux compared with predicted flux.** The growth physiological parameters used for pFBA simulation, as well as the 13C flux distribution of of *P. pastoris* G1HL was described in previous research (Lie et al. 2014).
