# Supplementary material for: Comprehensive reconstruction and evaluation of Pichia pastoris genome-scale metabolic model that accounts for 1243 ORFs
Source: Bioresour Bioprocess. 2017 May 9;4(1):22. doi: 10.1186/s40643-017-0152-x (PMC5423920; doi:10.1186/s40643-017-0152-x)
Supplement: Supplementary file 7 — Additional file 7. Measured and simulated influences of gene deletion or overexpression on the growth and S-adenosyl-l-methionine production by P. pastoris. [file 40643_2017_152_MOESM7_ESM.docx]

**Measured and simulated influences of** **gene deletion or overexpression on *P. pastoris* growth or S-adenosyl-L-methionin production.**

| **Strain background** | **Gene** | **Enzyme name** | **Manipulation** | ***In vivo*** | ***In silico*** | ***In vivo*** | ***In silico*** | **Reference** |
| --- | --- | --- | --- | --- | --- | --- | --- | --- |
|  |  |  |  | **Growth** | **Growth** | **Productivity** | **Productivity** |  |
| *G12'* | *vgb* | Vitreoscilla hemoglobin | inserting | - | - | ↑ | ↑ | Our lab |
|  | *spe2* | S-adenosylmethionin decarboxylase | Deletion |  |  |  |  |  |
| *G12'* | *gdh2* | Glutamate dehydrogenase | overexpressing | - | - | ↑ | - | Our lab |
| *G12'* | *zwf1* | Glucose-6-phosphate dehydrogenase | overexpressing | - | ↓ | ↑ | ↑ | Our lab |
| *G12'* | *sol3* | 6-phosphogluconolactonase | overexpressing | - | - | ↑ | - | Our lab |
| *G12'* | *mdh1* | Mitochondrial malate dehydrogenase | overexpressing | - | ↓ | - | - | Our lab |
| *G12'* | *gdh3* | Glutamate dehydrogenase | overexpressing | - | - | - | - | Our lab |
| *GS115* | *aox1* | S-adenosylmethionine synthetase 2 | inserting | - | ↓ | ↑ | ↑ |  |
| *GS115* | *cys4* | cystathionine-β-synthase | Deletion | - | - | ↑ | ↑ |  |
| *Gsam* | *cys4* | cystathionine-β-synthase | Deletion | - | - | ↑ | ↑ |  |
| *GS115* | *sam2* | S-adenosyl-L-methionine synthetase | overexpressing | ↓ | ↓ | ↑ | ↑ |  |
|  | *adk1* | adenylate kinase | overexpressing |  |  |  |  |  |

*MOMA was conducted with the specific S-adenosyl-L-methionin production rate as the objective function.

Note: －represents the manipulation has no direct effects on strain specific growth rate (growth) or specific S-adenosyl-L-methionin production rate (productivity), as well as the unreported results in the references. ↑ (↓) represents increase (decrease) in strain growth or productivity.

**References**

1. He, J., J. Deng, Y. Zheng and J. Gu (2006). "A synergistic effect on the production of S-adenosyl-L-methionine in Pichia pastoris by knocking in of S-adenosyl-L-methionine synthase and knocking out of cystathionine-beta synthase." J Biotechnol **126**(4): 519-527.
2. Li, D. Y., J. Yu, L. Tian, X. S. Ji and Z. Y. Yuan (2002). "[Production of SAM by recombinant Pichia pastoris]." Sheng Wu Gong Cheng Xue Bao **18**(3): 295-299.
3. Ravi Kant, H., M. Balamurali and S. Meenakshisundaram (2014). "Enhancing precursors availability in Pichia pastoris for the overproduction of S-adenosyl-L-methionine employing molecular strategies with process tuning." J Biotechnol **188**: 112-121.
